# Early Intervention in Severe Autism: Positive Outcome Using Exchange and Development Therapy

**DOI:** 10.3389/fped.2021.785762

**Published:** 2021-12-15

**Authors:** Romuald Blanc, Marianne Latinus, Marco Guidotti, Jean-Louis Adrien, Sylvie Roux, Pascale Dansart, Catherine Barthélémy, Aude Rambault, Frédérique Bonnet-Brilhault, Joëlle Malvy

**Affiliations:** ^1^Exac-T, Centre Universitaire de Pédopsychiatrie, CHRU de Tours, Tours, France; ^2^Université de Paris, Laboratoire de Psychopathologie et Processus de Santé, Boulogne Billancourt, France; ^3^UMR 1253, iBrain, Université de Tours, INSERM, Tours, France; ^4^Centro de Estudios en Neurociencia Humana y Neuropsicología, Facultad de Psicología, Universidad Diego Portales, Santiago, Chile

**Keywords:** autism spectrum disorder, children, assessment, Exchange and Development Therapy, Tailored and Inclusive Program for Autism-Tours

## Abstract

Early intervention programs positively affect key behaviors for children with autism spectrum disorder (ASD). However, most of these programs do not target children with severe autistic symptomatology associated with intellectual disability (ID). This study aimed to investigate the psychological and clinical outcomes of children with severe autism and ID enrolled in the Tailored and Inclusive Program for Autism—Tours (TIPA-T). The first step of the TIPA-T is the Exchange and Development Therapy (EDT): an individual neurofunctional intervention consisting of one-to-one exchanges between a child and a therapist taking place in a pared-down environment. It aims to rehabilitate psychophysiological abilities at the roots of social communication through structured sequences of “social play.” Cognitive and socio-emotional skills and general development were evaluated with the Social Cognitive Evaluation Battery scale and the Brunet–Lézine Scale—Revised, respectively, before and after 9 months of intervention in 32 children with ASD and ID. Autistic symptomatology was evaluated with the Behavior Summarized Evaluation—Revised scale at five time-points in a subset of 14 children, both in individual and group settings. Statistically significant post-intervention improvements were found in cognitive and socio-emotional skills. All but one child showed improvements in at least one social domain, and 78% of children gained one level in at least four social domains. Twenty-nine children improved in cognitive domains, with 66% of children improving in at least three cognitive domains. Autistic symptomatology evaluated in one-to-one settings significantly decreased with therapy; this reduction was observed in more than 85% of children. In group settings, autistic symptomatology also decreased in more than 60% of children. Global developmental age significantly increased by 3.8 months. The TIPA-T, including EDT in particular, improves socio-emotional skills of most children with ASD and reduces autistic symptomatology, yet with heterogeneous outcomes profiles, in line with the strong heterogeneity of profiles observed in ASD. At the group level, this study highlights the benefits of the TIPA-T for children with severe autism and associated ID. Assessment of autistic core symptoms showed an improvement of social interaction, both in one-to-one and group evaluations, demonstrating the generalizability of the skills learned during the EDT.

## Introduction

As defined in the Diagnostic and Statistical Manual of Mental Disorders, Fifth Edition (1), autism spectrum disorder (ASD) is a neurodevelopmental disorder characterized by impairments in social communication and social interaction and by restricted, repetitive patterns of behavior, interest, or activities that manifest during the first years of life. ASD is frequently associated with intellectual disability [ID, (2)], with a lower intellectual quotient linked to more severe autism symptoms (3). Most individuals with ASD and/or ID require some level of lifelong support because of the severity of these conditions and the high prevalence of related comorbidities. In addition, ASD is also characterized by an important heterogeneity, notably regarding the severity of autism but also at all levels of clinical examination: biological, genetic, cognitive, neural, and behavioral [e.g., (4, 5)]. Early intervention programs positively affect key behaviors for children with ASD. However, most of these programs do not target children with severe autistic symptomatology associated with intellectual disability. This study aimed to investigate the psychological and clinical outcomes of children with severe autism and intellectual disability enrolled in the Early Phase of the Tailored and Inclusive Program for Autism—Tours (TIPA-T).

Early intervention for children with ASD has been recognized as a health and educational priority (6, 7). Early (i.e., starting before 4 years old) intensive behavioral interventions are recognized as an efficacious approach for improving outcomes for young children with ASD (8–12). Well-known intervention programs based on a naturalistic developmental behavioral approach include the Early Start Denver Model (13), the Joint Attention Symbolic Play Engagement and Regulation (14, 15), Pivotal Response Treatment (16–18), Pediatric Autism Communication Therapy—Generalized (19), Frankfurt Early Intervention Program (20, 21).

Existing intervention programs mostly target children with no or mild intellectual deficiency. The TIPA-T program evaluated in the current study is dedicated to all children, from toddlers to young adults, including those with severe autism and associated intellectual disability.

The TIPA-T is a tailored and global program based on functional, developmental, and multidisciplinary assessments of the children. The program is set up by a multidisciplinary team, including psychiatrists, psychologists, speech therapists, psychomotor therapists, social workers, teachers, and nurses located in the child psychiatry intervention units in the Center of Excellence for Autism in Tours (EXAC-T) (France). The program is tailored to each child's age and needs following an integrative approach to the treatment of ASD and includes both individual and collective sessions. The current paper focuses on the early phase of TIPA-T, dedicated to children between 2 and 6 years old. The weekly program duration is 20 to 25 h following the recommendations of the High Authority for Health in France (HAS, 2012), integrating individual and collective care times [Exchange and Development Therapy (EDT), speech therapy, psychomotor therapy, and educative activities] and school sessions in mainstream kindergarten with individualized support.

Collective interventions, spread on the whole day, comprise speech and psychomotor activities performed by trained professionals, an educative program proposed by nurses and specialized teachers within the child psychiatric unit, alongside group free play aiming at working on socialization, communication, and autonomy. Collective interventions also integrate inclusive school sessions with individual support.

Individual intervention mainly consists of sessions of EDT, an individual neurofunctional therapy based on the experience and practice of a multidisciplinary team (22–24). The EDT aims at reeducating psychophysiological abilities at the roots of social communication, which will, in turn, improve behavior rather than target behavior first. The purpose of the EDT is not the child's performance but its participation in the proposed and shared activities. It focuses on developing, increasing, and enriching social contacts and exchanges with others through adapted means of communication. It is based on the underlying assumption that autistic symptoms are the consequences of the atypical development (25–29) and malfunctioning (25, 30–37) of the cerebral networks underlying change detection and social communication. The neurophysiological principles underlying EDT are cerebral plasticity, physiological curiosity, and free acquisition. It aims to rehabilitate, through structured sequences of “social play” and shared enjoyment, functions subtended by the brain systems of social communication: attention to others, intention, imitation, etc. The EDT consists of a one-to-one exchange between a child and a therapist taking place in a pared-down environment to facilitate mutual adjustments and socio-emotional synchronization between the child and the adult. This rehabilitation therapy is particularly indicated for young children before the age of 4 years, a period of maximum brain plasticity. The EDT is the pivotal element of the therapeutic and educational project built for a child in close relation with their family. Treatment organization is defined at the beginning of the session according to clinical and psychological assessments and behavioral deficits. Activities tailored to each child's needs and interests, evaluated before the therapy, and readjusted according to longitudinal evaluations are selected among a list of predefined activities (e.g., bubbles, motor games, mimed song, etc.).

A growing body of evidence on early interventions for children with ASD highlights a large variability in children's response to treatment [e.g., (38)]. However, so far, it is difficult to identify which children respond to which treatment making it difficult to recommend the intervention better suited to a specific child. Difficulty in assessing outcomes of intervention programs arose from the lack of clinical tools that provide a precise and detailed functional profile of children with autism, specifically oriented toward key symptoms of ASD and sensitive enough to assess subtle changes occurring over a very short period (38). In the current study, we used the Social Cognitive Evaluation Battery [SCEB; (39–42)], a French clinical tool created to assess young children with ASD with autism and associated ID. It explores different functional skills covering cognitive and socio-emotional domains for children with a developmental age (DA) comprised between 4 and 24 months. Complementary to the SCEB, the Behavioral Scale Evaluation—Revised [BSE-R; (32, 43)] evaluates the behavior of children with autism to further assess the severity of autism. Briefly, the BSE-R focuses on several neurophysiological functions, which are believed to contribute to core symptoms of autism in varying degrees (e.g., attention, perception, association, intention, imitation, contact, communication, etc.). The BSE-R provides a behavioral and functional profile of a child and can be used regularly to follow the evolution of a child's particular deficit (24, 32).

This study aimed to describe the evolution of key autistic symptoms and behaviors in some children with severe ASD and concurrent ID following a 9-month early intensive intervention program, the TIPA-T, centered on the EDT. We hypothesized that the TIPA-T would yield progress in socio-emotional skills assessed with the SCEB and a reduction of autistic behaviors with the BSE-R.

## Methods

### Subjects

The sample consisted of 32 children (26 males and 6 females) with a diagnosis of ASD, according to International Classification of Diseases 10 (World Health Organization, 1993) and Diagnostic and Statistical Manual of Mental Disorders, Fifth Edition (1), made by the multidisciplinary team of the Excellence Center for Autism—Tours after full clinical assessment. Mean chronological age was 45 ± [standard error of the mean (SEM)] 8.1 months (range in months [27 60]). All children had severe autism [Childhood Autism Rating Scale (44); mean ± SEM: 38.4 ± 0.51, (33.5 47)] and moderate-to-severe intellectual disability [Psychomotor Developmental Scale of Brunet–Lézine—Revised (45); developmental quotient: mean ± SEM: 39.3 ± 1.8, [15 60]; DA: 17.3 ± 0.7, [7 24]].

### Exchange and Development Therapy

The implementation of the EDT is highly structured by visual cues, visually based schedules, and the implementation of routines. EDT sessions take place two or three times a week and last approximately 20 min; sessions are always adapted to the child's attention and concentration skills.

The EDT is based on three general principles: serenity, availability, and reciprocity. The organization of EDT sessions aims at enforcing these general principles. To respect the principle of serenity, the EDT takes place in a bare room, thus creating a very sober space devoid of any distraction and precipitation. Before the session, the therapist prepares the room; that is, they choose furniture that will produce the best environment for inducing interactions with the child (a table and two chairs, or a mat on the floor and poufs) and the toys for the child. The aim is to create an environment that is stable from one session to another. The choice of toys is based on previous experience with the child; toys that have produced high-quality interactions at previous sessions are preferred.

To enforce the availability principle, the toys are offered one by one, in a predefined order, to keep the amount of stimulation to a minimum and focus the child's attention to the play at hand; nonetheless, the organization remains flexible to adapt to the child's envy. To keep the child involved in the sessions, both interactive and relaxing activities are proposed. This allows optimizing exchanges between the child and the therapist.

Perceptual-motor (e.g., mimed songs) and socio-emotional (e.g., itsy bitsy spider) sequences established around free play aimed at progressively increasing the synchronization between the child and the adult to promote reciprocity by fostering sociability. The therapist is continuously attentive to the child's communicative manifestations, however discreet they may be, to adapt the therapy to the child's reaction, and to include the child's initiatives in the sessions. If a child shows no interest in the proposed activity, the therapist tries gently to bring their attention back to the current activity by soliciting them gently. Importantly, they do so without showing disapproval to not reinforce the child's behaviour by a mark of attention. By doing so, the therapist manages to reengage the child and avoid a situation of failure. This permits that the child does not keep in mind a failed interaction. Finally, the adult promotes role-playing games and gradually introduces variations in the proposed scenari, considering the child's progress, so that established routines are not ritualized.

### Scoring

The Brunet–Lézine Scale—Revised (45) is an adaptation of Gesell's scales (46), validated in the French population. It allows the evaluation of the psychomotor development of children from 1 to 30 months of age. The Brunet–Lézine—Revised allows, in addition to the estimation of global developmental age (GDA) the assessment of developmental ages in four different areas: posture, oculo-manual coordination, language, and sociability (SDA).

Cognitive and socio-emotional skills were assessed with the Social Cognitive Evaluation Battery (40–42). Based on Piaget's, Bruner's, and Fisher's theories of psychological development (47), the SCEB assesses both cognitive and socio-emotional areas of development at four developmental levels (level 1 = from 4 to 8 months, level 2 = from 8 to 12 months, level 3 = from 12 to 18 months, and level 4 = from 18 to 24 months). The cognitive area comprised seven domains: self-image, symbolic play, object-relation schemata, operational causality, means–ends, spatial relations, and object permanence. The socio-emotional area includes nine domains: behavior regulation, social interaction, joint attention, expressive language, receptive language, vocal imitation, gestural imitation, affective relation, and emotional expression.

Autistic symptomatology was assessed using the BSE-R scale (32, 43). The BSE-R assesses 29 behaviors scored from 1 to 5 according to the frequency of occurrence (1 = never, 2 = sometimes, 3 = often, 4 = very often, and 5 = always). These 29 behaviors can be grouped into two factors: interaction deficits (factor 1) and modulation deficits (factor 2). Here, the data of each factor were analyzed. The BSE-R scale is a recognized tool used in observational and intervention evaluation studies (48).

### Organization of Assessments

Scorings of the Brunet–Lezine scale and the SCEB were performed at the beginning of therapy and after 9 months by psychologists experienced with these tools. The initial evaluation necessary to work out a tailored therapy program constitutes the baseline. The BSE-R scale was evaluated both in a one-to-one therapy session, through recordings of EDT sessions, and in collective settings to study a generalization of acquired skills at months 1, 3, 5, 7, and 9 of therapy.

### Data Analysis

Statistical analysis and figures were made with R [version 4.0.2; (49)] within Rstudio [version 1.3.1056; (50)] environment using the following packages: lmerTest (51), rstatix (52), ggplot2 (53), tidyverse (54), readr (55), readxl (56), ggpubr (57), ggiraphExtra (58), and ggradar (59).

**Figure 1:**
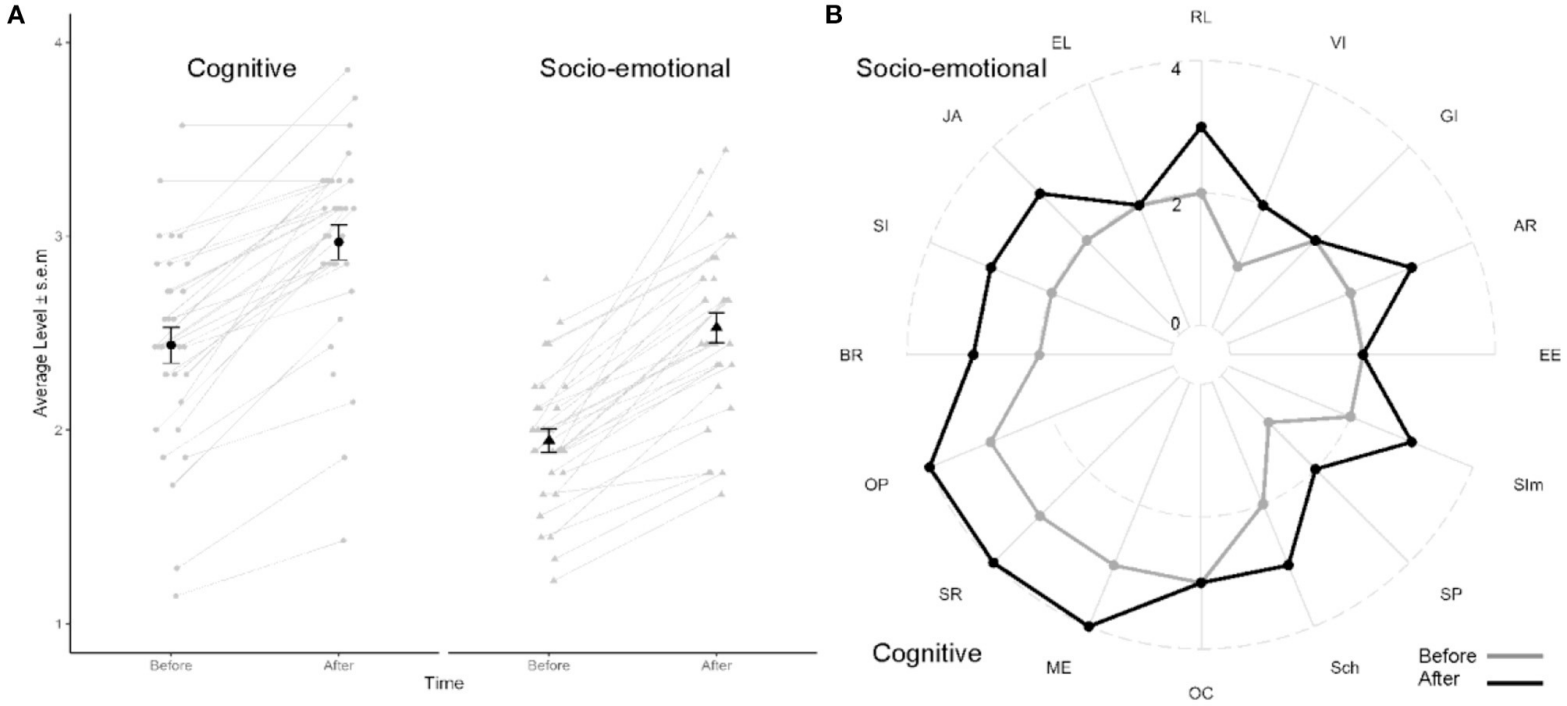
Cognitive and socio-emotional assessment. **(A)** Average level across all cognitive and all socio-emotional domains before and after 9 months of therapy. Each individual is plotted as a gray line. Black dots represent group mean ± standard error of mean. Note that participant 28 was the participant producing the lowest scores; however, a progression was also observed with therapy. **(B)** Radar plot of statistical mode for each domain assessed with SCEB. Socio-emotional are presented on top and cognitive domains on bottom of image. Grayline, before therapy; black line, after therapy. Socio-emotional domains: BR, behavior regulation; SI, social interaction; JA, joint attention; EL, expressive language; RL, receptive language; VI, vocal imitation; GI, gestural imitation; AR, affective relation; EE, emotional expression. Cognitive domains: SIm, self-image; SP, symbolic play; Sch, object-relation schemata; OC, operational causality; ME, means–ends; SR, spatial relations; OP, object permanence.

**Figure 2:**
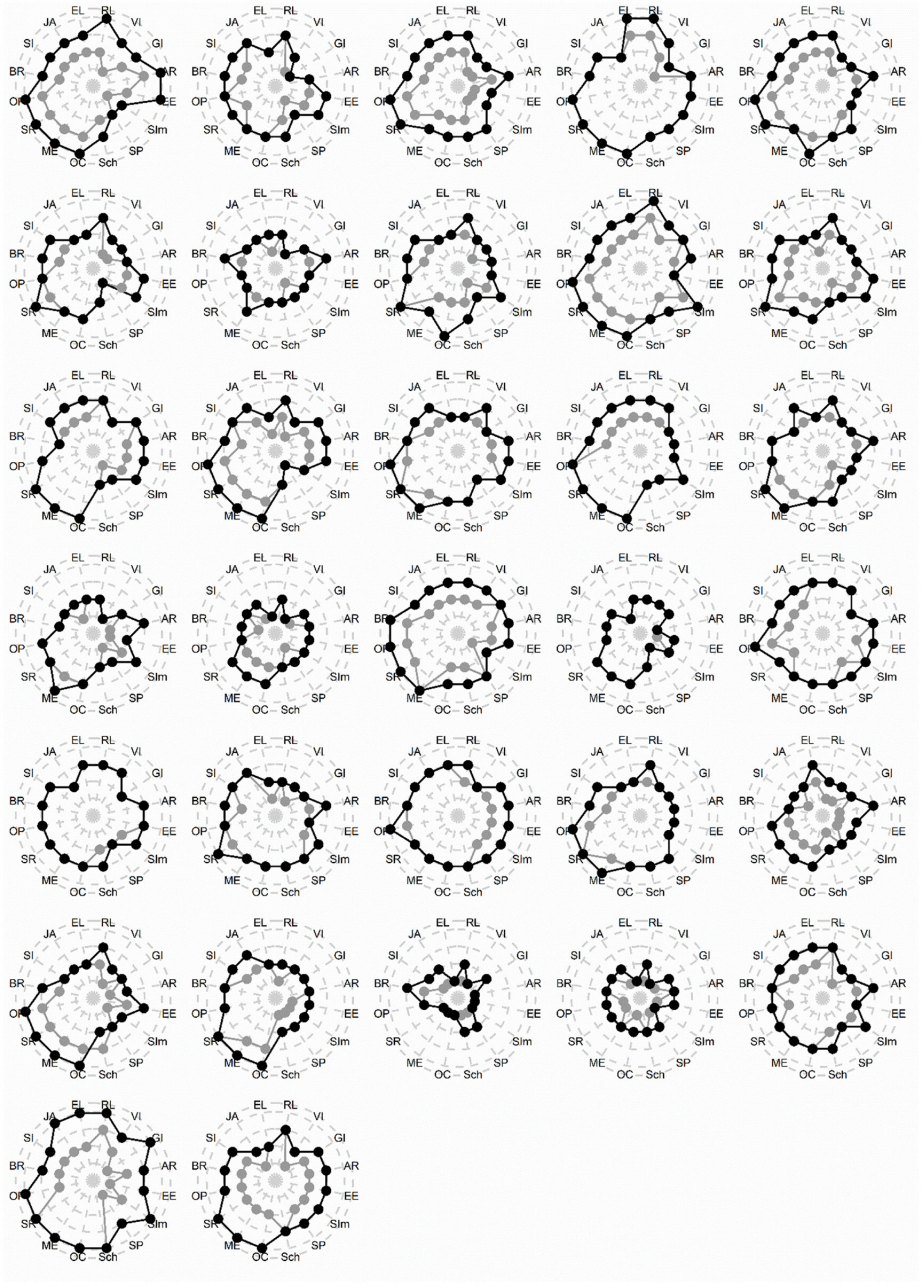
Individual data (*N* = 32) for cognitive and socio-emotional assessment. Each radar plot represents a child enrolled in TIPA-T. Grayline: before therapy; black line: after therapy. Note that Subject 28 (before last line, middle column) was an extreme outlier and was removed from statistical analysis. Socio-emotional domains (top): BR, behavior regulation; SI, social interaction; JA, joint attention; EL, expressive language; RL, receptive language; VI, vocal imitation; GI, gestural imitation; AR, affective relation; EE, emotional expression. Cognitive domains (bottom): SIm, self-image; SP, symbolic play; Sch, object-relation schemata; OC, operational causality; ME, means–ends; SR, spatial relations; OP, object permanence).

SCEB scores averaged across all social and all non-social domains (Figure 1A) were analyzed with a two-way repeated measure analysis of variance (ANOVA) with time (before/after therapy) and domains (socio-emotional/cognitive) as within-subject factors. Previous to the ANOVA, assumptions were verified, and participant 28 (Figure 2) was identified as an extreme outlier {outside the range [Q1 – 3 ^*^ interquartile range (IQR) Q3 + 3 ^*^ IQR]; in cognitive domains after therapy} using the IQR method: his averaged SCEB scores were all outside the range (Q1 - 1.5 ^*^ IQR Q3 + 1.5 ^*^ IQR) (lowest values on Figure 1A). Shapiro–Wilk tests performed on each factor combination from the 31 remaining participants highlights that the data did not differ from a normal distribution (*p* > 0.09); before removing participant 28, data in the cognitive domain after therapy did not follow a normal distribution (*p* = 0.01).

**Figure 3:**
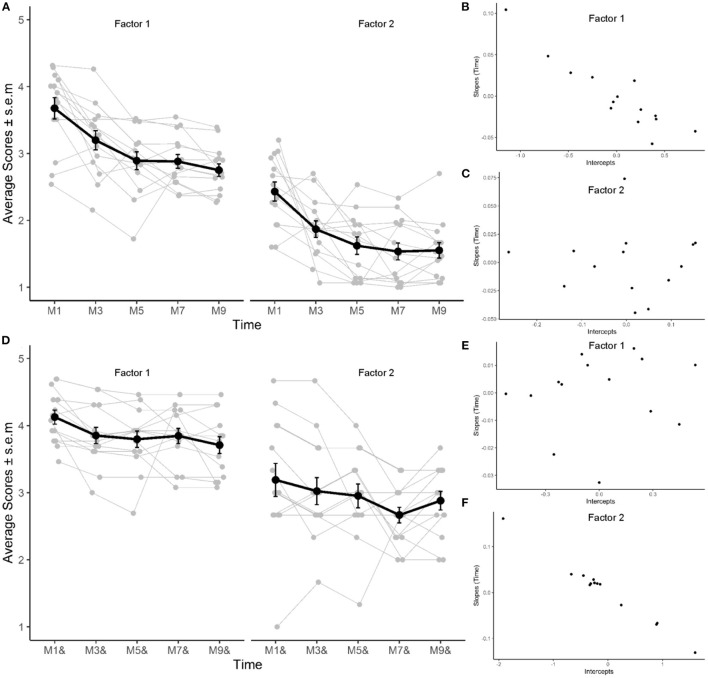
Evolution of factors 1 and 2 of BSE-R during therapy. **(A–C)** Evaluation performed after one-to-one exchanges. **(D–F)** Evaluation performed in group therapy. **(A,D)** Average (black) and individual (gray; *N* = 14) scores assessed at months 1, 3, 5, 7, and 9 of therapy. **(B,E)** Correlation of within-subjects random intercept and slopes for factor 1 in individual and group therapy session, respectively. **(C,F)** Correlation of within-subjects random intercept and slopes for factor 2 in individual and group therapy session, respectively.

For each factor of the BSE-R, longitudinal scores were fitted with a linear mixed model with two fixed-effects parameters, intercept and slope, of the linear trend over time (e.g., month of therapy) for the population and two random effects for each subject. Random effects for a particular subject were the deviations in intercept and slope of that subject's time trend from the population. The model allowed for the correlation of random effects for the same subject due to the relationship between intercept and slope (Figure 3). The model was fit as: scores ~ 1 + time + (1 + time | subject). *T*-tests for fixed effects parameters use the Satterthwaite's method for the degree of freedom calculation as in lmerTest (51).

Developmental assessments using Brunet–Lézine data were analyzed at the beginning and at the end of therapy. GDA was analyzed using a paired Student *T*-test after removing one extreme outlier (participant 31 who showed an overall improvement of 13 months); effect size was computed as Cohen's d. DAs for each area were analyzed with Wilcoxon signed-ranked test on paired data. Participant 32 was identified as an extreme outlier in SDA, showing an improvement of 19 months, and was therefore removed for SDA analysis.

## Results

### Cognitive and Socio-Emotional Assessment

A repeated measure ANOVA with time and domain as within-subject factor revealed main effects of time [F_(1,30)_ = 125.89, *p* < 0.001, η^2[g]^ = 0.32] and domains [F_(1,30)_ = 97.18, *p* < 0.001, η^2[g]^ = 0.26] on the average levels of the SCEB but no interaction [F_(1,30)_ = 0.85, *p* = 0.36, η^2[g]^ <0.001; Figure 1A]. Average levels were higher for cognitive than socio-emotional domains and were higher after 9 months of therapy. For information, an ANOVA with all participants yielded similar results, with effects of time [F_(1,31)_ = 129.24, *p* < 0.001, η^2[g]^ = 0.27], area [F_(1,31)_ = 80.86, *p* < 0.001, η^2[g]^ = 0.21], and no interaction.

At the beginning of therapy, the developmental level was low for both socio-emotional [mean level across the different domains: 1.94 ± 0.06; (1.2 2.8)] and cognitive domains [2.44 ± 0.09; (1.1 3.6)]. After 9 months of therapy, mean levels had increased by approximately 0.5 for both social [2.53 ± 0.08; (1.7 3.4)] and cognitive [2.97 ± 0.09; (1.4 3.9)] domains (Figure 1A). To have a more precise comprehension of the evolution of children's profiles with therapy, descriptive statistics using statistical mode were used (Figure 1B). The statistical mode, corresponding to the level shown by most children, increased by one level with therapy for all cognitive domains but operational causality. For skills in the social domains, a one-level increment was observed for behavioral regulation, social interaction, joint attention, receptive language, vocal imitation, and affective relation.

Exploration of individual data (Figure 2) revealed a majority of children gained at least one level in all the socio-emotional domains: behavioral regulation, social interaction, joint attention, receptive language, vocal imitation, affective relation, and emotional expression (Figure 2). In cognitive domains, a majority of children improved in self-image, symbolic play, spatial relation, and object permanence.

All but one child showed improvements in at least one social domain, and 78% of children gained one level in at least four social domains. Twenty-nine children improved in cognitive domains, with 66% of children improving in at least three cognitive domains.

### Behavioral Assessment

Exploration of the interaction and modulation deficits assessed with the BSE-R in individual EDT session revealed an average reduction of symptoms of 0.92 and 0.88, respectively. Attenuation of interaction deficits was observed in 93% of children, and modulation deficits decreased in 86% of children.

For the interaction deficits factor, estimated fixed parameters (β) for time, across the population, were significant for both intercepts {β [95% confidence interval (CI)]: 3.6 [3.3 3.9]; T_(14)_ = 22.3; *p* < 0.0001} and slope [β (95%CI):−0.11 (-0.15−0.07); T_(14)_ = −5.8; *p* < 0.0001], highlighting that interaction deficits before therapy were high but decreased by approximately 0.1 point (on a five-point scale) per month of therapy (Figure 3A). The standard deviation of random effects for intercept and slope were 0.54 and 0.05, respectively, suggesting that in a typical population, expected factor 1 scores would vary between 2.55 and 4.65 and that the expected decrease due to therapy would vary between−0.2 and−0.002. In addition, a high within-subject correlation between random effects for intercept and slope (−0.87; Figure 3B) for factor 1 was observed, highlighting a strong relationship between severity of interaction deficits at the start of therapy and decrease in autistic symptomatology in the population: the larger the deficits at the start of therapy, the larger was the improvement brought on by the therapy.

For the modulation deficits, estimated fixed parameters for time were significant for intercept [β (95%CI): 2.3 (2.1 2.6); T_(14)_ = 20.2; *p* < 0.0001] and for slope [β (95%CI):−0.1 (-0.15−0.06); T_(14)_ = −4.95; *p* = 0.0002; Figure 3A], revealing that typical modulation deficits (F2) in the population were less high than interaction deficits at the start of therapy (2.3) but still decreased by approximately 0.1 point per month of therapy. The standard deviation of random effects for intercept and slope were 0.21 and 0.04, respectively, suggesting that in a typical population, expected factor 2 scores would vary between 1.92 and 2.75 and that the expected decrease due to therapy would vary between−0.18 and−0.03; random effects for slope and intercept were less correlated (correlation coefficient: −0.45; Figure 3C).

Exploration of the interaction and modulation deficits assessed with the BSE-R in group sessions revealed an average reduction of symptoms of 0.42 and 0.31, respectively. Attenuation of interaction deficits was observed in 86% of children, and modulation deficits decreased in 64% of children.

For BSE-R evaluated in group therapy, effect of time was significant for both intercept [β (95%CI): 4.1 (3.8 4.3); T_(14)_ = 37.2; *p* < 0.0001] and slope [β (95%CI):−0.04 (-0.07−0.01); T_(14)_ = −3.3; *p* = 0.0057] for factor 1 (Figure 3D), highlighting a decrease of interaction deficits of approximately 0.04 per month of therapy. Correlation coefficient for within-subjects' random effects of intercept and slope was−0.19 (Figure 3E). Effect of time for factor 2 (Figure 3D) evaluated in group therapy sessions was significant for intercept [β (95%CI): 3.2 (2.7 3.7); T_(14)_ = 12.8; *p* < 0.0001] but not for slope [β (95%CI):−0.05 (-0.1 0.003); T_(14)_ = −1.98; *p* = 0.07]. Correlation coefficient for within-subjects' random effects of intercept and slope was high (-0.98; Figure 3F).

### Developmental Assessment

**Figure 4:**
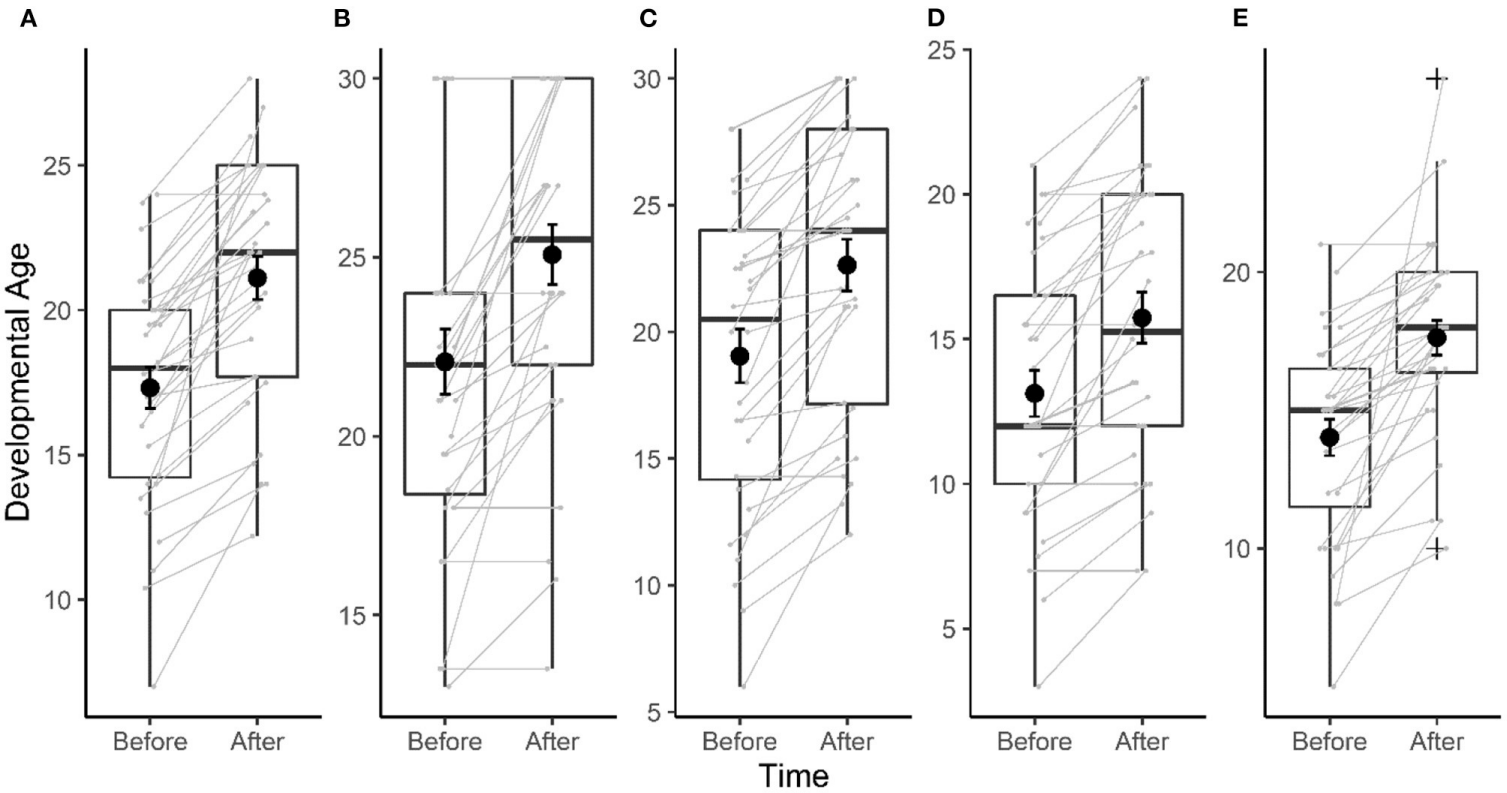
Evolution of developmental age assessed with Brunet–Lézine before and after 9 months of therapy. **(A)** Global developmental age (GDA). **(B)** Postural developmental age (PDA). **(C)** Oculo-manual coordination developmental age (CDA). **(D)** Language developmental age (LDA). **(E)** Sociability developmental age (SDA). Black dot and error bar represent mean and SEM. Gray dot and connecting lines represent individual data.

GDA assessed with the Brunet–Lézine scale significantly increased by an average of 3.8 months with therapy [GDA, T_(30)_ = 10.15, *p* < 0.001, Cohen's d = 1.5; Figure 4A]. Of the 32 children included in the study, only one child showed no increase in their GDA.

An improvement of approximately 3 months was also found in each area assessed with the Brunet–Lezine with large effect sizes (*r* > 0.7): postural developmental age (*V* = 210, *p* < 0.001, *n* = 32, *r* = 0.77; Figure 4B), oculo-manual developmental age (*V* = 465, *p* < 0.001, *n* = 32, *r* = 0.87; Figure 4C), language developmental age (*V* = 300, *p* < 0.001, *n* = 32, *r* = 0.82; Figure 4D), and SDA (*V* = 465, *p* < 0.001, *n* = 31, *r* = 0.87; Figure 4E). A large majority of children showed an improvement of their DA over the 9 months of therapy in each area, with the smallest proportion observed for the postural developmental age (62.5% of children).

Keeping outliers in the statistical analysis does not change observed effects: GDA: T_(31)_ = 8.49, *p* < 0.001, Cohen's d = 1.5, SDA: *V* = 465, *p* < 0.001, *n* = 32, *r* = 0.87.

## Discussion

Using clinical tools tailored for ASD and sensible to subtle changes occurring over a short amount of time (the SCEB and the BSE-R), positive outcomes were observed in children with severe ASD and concurrent ID following a 9-month early intensive intervention program, the TIPA-T centered on the EDT in a specialized medical center. The TIPA-T, including its pivotal first step, the EDT, allowed for a general improvement for both cognitive and socio-emotional domains and a reduction in the severity of autistic symptomatology. In addition, assessment of autistic core symptoms with the BSE-R showed a decrease of interaction deficits, in particular, both in one-to-one and in group evaluations. The latter demonstrates the generalizability of the skills learned during the EDT.

Children's developmental trajectories were positive, but their evolution was atypical and characterized by uneven progress. Large individual variations in outcomes were observed in this study, consistent with the findings from previous research (10, 20, 21, 24, 60) and in line with the strong heterogeneity of profiles observed in ASD. However, general improvement for each cognitive and socio-emotional domain was seen during therapy (e.g., joint attention, imitation, social interaction, etc.). Moreover, autistic behaviors tended to decrease (reduction in the degree of severity of the deficits in interaction and modulation), particularly in one-to-one exchanges. Although we cannot be certain that these results do not reflect the natural maturation of children, we believe this is unlikely because we observed progress in key domains of autistic symptomatology, specifically targeted by the EDT. Moreover, poor outcomes were observed in some children suggesting that natural maturation alone is unlikely to improve performance. Finally, in the current study, no children showed a worsening of their condition within the 9 months of the therapy, whereas a 9% worsening rate could have been expected based on natural maturation only (61).

Results of this study are consistent with other follow-up studies showing that early and intensive intervention program over a relatively short period yields positive outcomes for children with autism. Dawson and collaborators (13) showed the efficacy of an intensive intervention program designed for toddlers with ASD as young as 18 months: the Early Start Denver Model (ESDM). After 2 years of intervention, children provided with the ESDM showed significant improvements in IQ, adaptive behavior, and diagnostic status. More recently, it was shown that low-intensity ESDM is of some benefit to children with ASD in imitation, engagement, and intentional vocalizations (62). The results of a 1-year study on a developmentally based social pragmatic approach, the Frankfurt Early Intervention program, which starts on average at 66 months, showed improvements in autistic symptoms and cognitive development (20, 21). Pivotal Response Treatment, in children aged 64 months on average, results in increases in self-initiations and has positive effects on interaction and verbal communication, play skills, and maladaptive behavior for a number of children (17, 18). In line with these studies, the developmental and behavioral progress of children included in the TIPA-T program, which are generally more retarded, were important for a short period. Although the current study measures improvements following EDT in slightly older children than the ESDM, the EDT is also designed for younger children and children with ASD and severe ID, and the program can be started before age 2 years. The EDT relies on a functional baseline, which not only allows characterizing the changes brought on by the therapy but also helps to target and prioritize specific functions, making it an essential therapeutic tool for children with ASD and with specific needs. Consistently, the EDT benefits severe more to children with severe autistic symptomatology, for whom the progression was larger. Taking together these studies demonstrate that when therapy is provided systematically for children with ASD within the framework of highly structured and intensive therapy, contact behaviors, exchange, and communication deficits are reduced both in the short and long terms (23, 31, 63–67). Improvements also affect both “primary” behavioral disturbances such as disorders of perception and association and “secondary” symptoms such as social withdrawal (22).

Difficulties in assessing the benefits of intervention programs arose from the lack of clinical tools dedicated to assessing subtle changes occurring over a small period (38). Here, we used the SCEB (39–42), which allows assessing a range of cognitive and socio-emotional skills for young children with ASD associated with a moderate-to-severe intellectual disability. The BSER-R (32, 43) provides additional information regarding the severity of autistic symptomatology, focusing on key behaviors contributing to core symptoms of ASD in varying degrees (e.g., attention, perception, association, intention, imitation, contact, communication, etc.). The use of these scales, which allows measurement of subtle changes, revealed progress in different cognitive and socio-emotional domains and a reduction in autistic symptomatology, even over a period as short as 9 months. Moreover, the BSE-R can be evaluated in one-to-one and in group sessions, allowing the demonstration of generalization of the skills learned during the EDT, in particular regarding interaction deficits. Finally, using these scales helped identify the functions that were the first to respond to therapy and the resistant ones, providing orientations for therapy prevention and early intervention (11, 20, 24, 35). Combining autistic behaviors, cognitive and socio-emotional abilities assessments provide the basis for a richer dialogue with families to improve educational and therapeutic synergy around the child. It also meets specific needs to specify and individualize the contents of psycho-educational and therapeutic actions, making them essential batteries for child psychiatric teams in daily practice.

Using these assessments complemented the findings obtained with a classical tool such as the Brunet–Lézine—Revised scale. Results from the Brunet–Lézine—Revised scale showed a general improvement in DA, as well as domain-specific improvement. On average, the 9-month therapy yielded a 3-month gain in DA, and importantly, this increase in DA was observed in a large majority of children. This highlights the benefit of the TIPA-T, including specifically the EDT for the cognitive development of children considering that children with low IQ tend to maintain their IQ over time (61), consistent with previous observations that intellectual disability in children with ASD who have benefited from early intensive care can be improved (9, 13, 68, 69). However, the Brunet–Lezine scale is a non-specific clinical tool that, despite allowing only for assessing the evolution of DAs and quotients, can be used to monitor children with ASD. The results of the current study showed that with more specialized and precise tools such as the SCEB and BSE-R, the child could be understood as a whole, and clinicians can obtain subtler and more precise information regarding the effects of treatment in children with ASD (38).

There are several important clinical implications for these data. When diagnosing young children with ASD, it is important to assess cognitive skills and social-reciprocal interaction deficits and abilities using standardized tests. Both measures are strongly related to outcomes and are correlated with each other. These measures can help clinicians to assess responsiveness to intervention and treatment planning. In addition, early social-interaction abilities may be a pivotal skill that should be addressed in intervention programs. The study also emphasizes the effectiveness of the intervention at a very early age across the autistic symptomatology severity range. A therapy such as the EDT might also benefit from being developed in at-home settings by providing support and teaching parents the functional basis and the implementation of EDT at home.

## Conclusions

The TIPA-T, including specifically EDT, improves cognitive and social skills and core symptoms of autism of most children with severe ASD and associated ID. Assessment of autistic core symptoms with the BSE-R showed a decrease of interaction disorder in particular, both in one-to-one and group evaluations. Importantly, this demonstrates the generalizability of the skills learned during the EDT. However, as for other intervention programs in autism, large individual variations were seen in the outcomes, in line with the strong heterogeneity of profiles observed in ASD. Data acquired in the current study do not allow understanding the reasons for the therapy being extremely successful in some children and less in others. Future studies should aim at identifying predictive factors of success to potentiate positive outcomes.

## Data Availability Statement

The raw data supporting the conclusions of this article will be made available by the authors, without undue reservation.

## Ethics Statement

The studies involving human participants were reviewed and approved by CHRU Tours. Written informed consent to participate in this study was provided by the participant's legal guardian/next of kin.

## Author Contributions

CB, J-LA, and RB contributed to conception and design of the study. RB, MG, JM, PD, and AR contributed to data collection. SR organized the database. ML performed statistical analyses. RB, ML, JM, FB-B, and J-LA wrote the first draft of the manuscript. All authors contributed to manuscript revision, read, and approved the submitted version.

## Conflict of Interest

J-LA is the author of the SCEB edited in the Pearson France-ECPA. The remaining authors declare that the research was conducted in the absence of any commercial or financial relationships that could be construed as a potential conflict of interest.

## Publisher's Note

All claims expressed in this article are solely those of the authors and do not necessarily represent those of their affiliated organizations, or those of the publisher, the editors and the reviewers. Any product that may be evaluated in this article, or claim that may be made by its manufacturer, is not guaranteed or endorsed by the publisher.

## Abbreviations

ASD: Autism Spectrum disorder
TIPA-T: Tailored and Inclusive Program for Autism—Tours
EDT: Exchange and Development Therapy
SCEB: Socio-Emotional and Cognitive Evaluation Battery
BSE-R: Behavioral Scale Evaluation—Revised.
